# Clinical and psychological factors in coronary heart disease patients with statin associated muscle side-effects

**DOI:** 10.1186/s12872-021-02422-7

**Published:** 2021-12-16

**Authors:** Kari Peersen, John Munkhaugen, Elise Sverre, Oscar Kristiansen, Morten Fagerland, Nils Tore Vethe, Joep Perk, Einar Husebye, Toril Dammen

**Affiliations:** 1 Department of Medicine, Vestfold Hospital Trust, Tønsberg, Norway; 2 Department of Behavioral Medicine and Faculty of Medicine, University of Oslo, Oslo, Norway; 3 Department of Medicine, Drammen Hospital, Drammen, Norway; 4 Oslo Centre for Biostatistics and Epidemiology, Research Support Services, Oslo University Hospital, Oslo, Norway; 5 Department of Pharmacology, Oslo University Hospital, Oslo, Norway; 6 Faculty of Health Sciences, Linnaeus University, Kalmar, Sweden

**Keywords:** Statin-associated muscle symptoms, Coronary heart disease, Psychological factors, Beliefs in medicine, Atorvastatin, Crossover trial, Placebo-controlled

## Abstract

**Background:**

To compare clinical and psychological factors among patients with self-perceived statin-associated muscle symptoms (SAMS), confirmed SAMS, and refuted SAMS in coronary heart disease patients (CHD).

**Methods:**

Data were obtained from a cross-sectional study of 1100 CHD outpatients and a study of 71 CHD outpatients attending a randomized, double-blinded, placebo-controlled, crossover study to test effects of atorvastatin 40 mg/day on muscle symptom intensity. Clinical and psychosocial factors were compared between patients with and without SAMS in the cross-sectional study, and between patients with confirmed SAMS and refuted SAMS in the randomized study.

**Results:**

Bilateral, symmetric muscle symptoms in the lower extremities during statin treatment were more prevalent in patients with confirmed SAMS compared to patients with refuted SAMS (75% vs. 41%, *p* = 0.01) in the randomized study. No significant differences in psychological factors (anxiety, depression, worry, insomnia, type D personality characteristics) were detected between patients with and without self-perceived SAMS in the cross-sectional study, or between patients with confirmed SAMS and refuted SAMS, in the randomized study.

**Conclusions:**

Patients with confirmed SAMS more often present with bilateral lower muscle symptoms compared to those with refuted SAMS. Psychological factors were not associated with self-perceived SAMS or confirmed SAMS. A careful pain history and a search for alternative causes of muscle symptoms are likely to promote communication in patients with SAMS, and may reduce the risk for statin discontinuation.

## Background

Self-perceived statin-associated muscle symptoms (SAMS) cover a heterogeneous group of muscle symptoms including pain, aching, stiffness, tenderness or cramps [1]. SAMS, reported in 10–25% of patients using statins, is a principal reason for dose reduction, non-adherence and discontinuation [1]. More knowledge about SAMS is needed to develop new strategies to prevent the loss of effective statin therapy in patients with coronary heart disease (CHD) [1, 2].

Previous research has mainly focused on the pathophysiologic mechanisms underlying SAMS [1]. Even though associations between psychological distress and SAMS have been proposed [3], studies are scarce. Anxiety has been associated with increased pain perception [4], increased risk of misattributing muscle symptoms to statins or being hypervigilant to true adverse effects of statins such as muscle pain [5], and thus may render a patient more susceptible to SAMS. On the contrary, a randomized controlled atorvastatin trial in patients without cardiovascular disease, did not find distress in terms of higher levels of depression to predict changes in muscle pain severity between the atorvastatin and placebo group after 6 months follow-up [3]. Other psychological factors with potential associations to increased pain perception, musculoskeletal pain and more widespread pain syndromes are distressed (i.e. type D) personality and insomnia [6–9]. Together, these results suggest that various psychological factors may influence self-perception of pain and contribute to a self-report of SAMS. If so, we may hypothesize that among statin users, those who report SAMS would have higher levels of these psychological factors compared to those who do not report SAMS.

Although clinical diagnostic algorithms for SAMS have been proposed, a gold standard definition is yet to be established. Two recent randomized crossover trials have documented that self-perceived SAMS is not related to statin intake in most patients [10, 11]. In a randomized double-blinded crossover trial, we classified 71 CHD patients with self-perceived SAMS into 20 with confirmed SAMS (i.e. statin-dependent) and 51 with refuted SAMS (i.e. statin-independent) by exposing participants to 7-weeks treatment with atorvastatin 40 mg/day and matched placebo in a random order [12]. Altogether, these results suggest that the majority of those who report SAMS have muscle complaints unrelated to statin use. The relationship between psychological factors within the SAMS group are intriguing as this population includes two subgroups, patients with true SAMS, defined as confirmed SAMS, and patients with muscle complaints unrelated to statins, defined as refuted SAMS. Psychological factors may differ between these two subgroups. To the best of our knowledge, we do not know of any previous study that has compared characteristics of patients with confirmed SAMS to patients with refuted SAMS within the group with SAMS. New insight into the relationship between psychological factors and muscle symptoms in patients with self-perceived SAMS in general and in those with confirmed SAMS in particular may aid in the identification of risk factors for SAMS, as well as distinguishing those who are most likely to have unspecific muscle complaints from those with confirmed SAMS. This may have implications for the clinical management of these patients and may reveal alternative explanations for the patients’ muscle complaints that should be addressed.

Patients’ beliefs about their medical treatment, including necessity and concerns, is another determinant with potential associations to statin side-effects and adherence [13]. In patients surviving an acute coronary syndrome, greater perceived necessity and lower perceived concern about statins were independently associated with statin adherence [14]. However, the relationship between patients’ beliefs, SAMS and adherence was not assessed. The relationship between patients’ beliefs about statin therapy and SAMS has yet not been investigated.

The clinical presentation, including location, intensity and characteristics of muscle symptoms in patients with SAMS, is heterogeneous reflected by the variety of definitions in literature [1]. SAMS have been described to appear bilateral, symmetrically affecting the large proximal muscles, particularly of the lower extremities [15, 16]. The clinical presentation of muscle symptoms in patients with and without confirmed SAMS remains to be investigated. Such knowledge may further aid in distinguishing those with true SAMS from those with independent muscle complaints.

This pre-planned sub-study aim to [1] compare clinical and psychological factors in CHD patients with and without self-perceived SAMS in a cross-sectional study and [2] compare psychological factors, beliefs about statin use and muscle symptom presentation between patients with confirmed SAMS and refuted SAMS in a randomized study.

## Methods

### Design and study populations

**Fig. 1:**
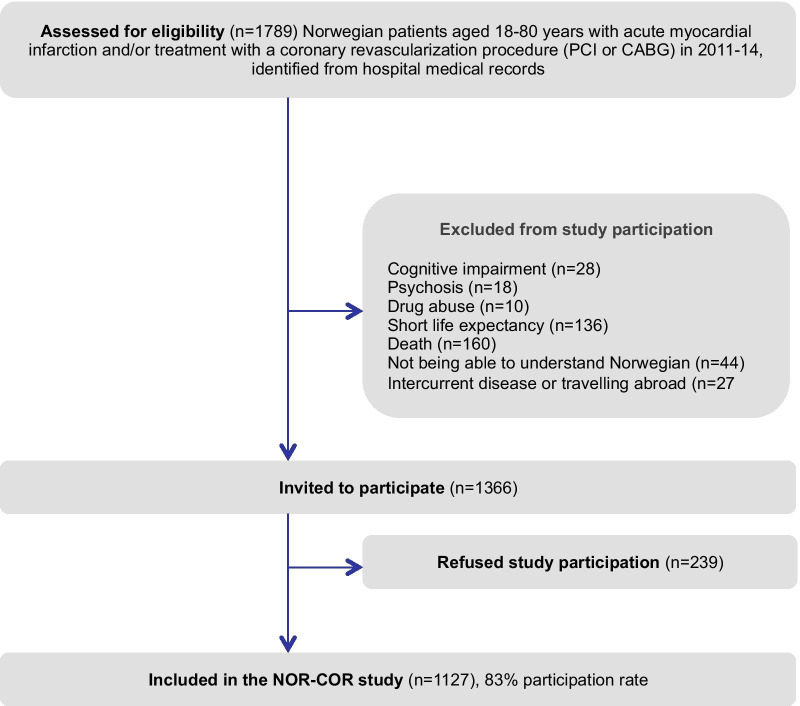
The NORCOR study flowchart

Data were obtained from two independent patient samples. Data from the NORwegian CORonary prevention (NORCOR) study was used for the first aim, Fig. 1 the NORCOR study flowchart. The design of the NORCOR study has been described elsewhere [17, 18]. Briefly, this is a cross-sectional study including 1127 consecutive patients, aged 37–80 years from two general hospitals (Drammen and Vestfold) in Norway. All patients were hospitalized with acute myocardial infarction (80%) and/or a coronary revascularization procedure in 2011–14. The patients attended a clinical visit including blood samples and completed a comprehensive questionnaire 2–36 (median 16) months after the index event. A broad time range from index event to inclusion was chosen to enlarge the sample size and to be able to assess changes in risk factor control with increasing time since the coronary event. Many of the included items and instruments in the questionnaire were previously validated [17]. We also performed a reproducibility test of the NORCOR study questionnaire with highly acceptable result [19]. Data on side-effects of cardiovascular drugs were missing in 27 patients, thus 1100/1127 (97.6%) were included in the present study. In all, 70 (6.4%) patients reported SAMS.

**Fig. 2:**
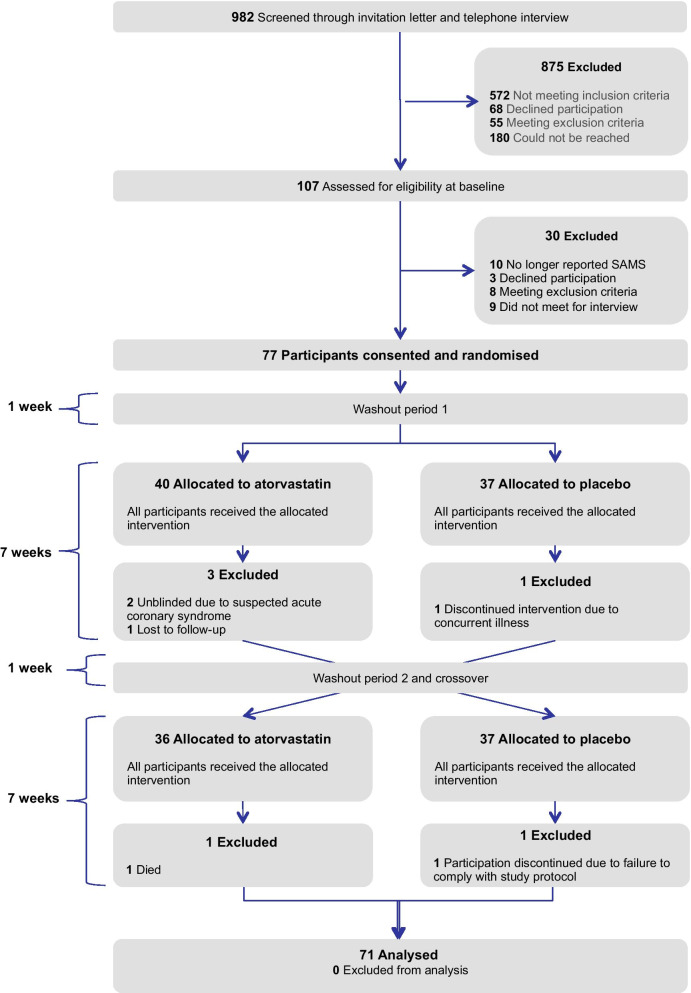
The Muse trial flowchart

Data from the MUscle Side-Effects of atorvastatin in coronary patients (MUSE) trial was used for the second aim, Fig. 2 the MUSE trial flowchart [20]. MUSE was a 16 week randomized, double-blinded, placebo controlled, two period AB/BA crossover study to test the effect of atorvastatin 40 mg/day on muscle symptom intensity. The study design and methods are described elsewhere [20]. Consecutive patients with a first or recurrent myocardial infarction hospitalized at Drammen and Vestfold between 2016 and 2019 were retrospectively identified through discharge lists and a standardized telephone interview. All patients with self-reported on-going or previous atorvastatin-associated muscle symptoms were invited to the outpatient clinics for a final evaluation of the entry criteria and randomization. A detailed description of screening procedure, including inclusion and exclusion criteria is provided elsewhere [12]. The questionnaire used in the MUSE trial was almost equal to the NORCOR study questionnaire, supplemented with validated scales described in the covariate paragraph [20]. In all, 71 patients, classified into 20 (28%) with confirmed SAMS (i.e. statin-dependent) and 51 (72%) with refuted SAMS (i.e. statin-independent), completed the study.

### Outcome assessment

Self-perceived statin side-effects in NORCOR study: Statin side-effects were reported on the study questionnaire qualitatively and categorized as SAMS by two different cardiologists [18].

Confirmed SAMS and refuted SAMS in the MUSE trial: Muscle symptom intensity was registered at the time of randomization and weekly during the treatment periods in a diary using a zero (no symptoms) to 10 (worst imaginable) cm visual-analogue scale (VAS). Confirmed SAMS was predefined as a 25% higher individual mean VAS-score during 7-weeks treatment with atorvastatin 40 mg/day versus 7-weeks treatment with placebo, and ≥ 1 cm absolute difference, as suggested in a validation study [21]. Those who were not categorized as confirmed SAMS were categorized as refuted SAMS. The mean VAS-score was analysed on the basis of muscle symptom intensity in the last three weeks of each treatment period.

### Covariates

The following socio-demographic, clinical and psychosocial factors were collected from hospital records, a self-report questionnaire and clinical examination at study inclusion in the cross-sectional NORCOR study [17] and at the time of randomization in the MUSE trial [20]: marital status, level of education, medical history (cardiovascular comorbidity and muscle-skeletal complaints), current treatment with lipid lowering drugs, statin adherence and discontinuation, time since index event, physical activity, smoking habits, weight and height, Hospital Anxiety and Depression Scale (HADS), Type D distressed personality Scale (DS-14), Penn State Worry Questionnaire (PSWQ) and Bergen Insomnia Scale.

The following covariates were collected in the MUSE trial only: Beliefs in statin therapy; The Beliefs about Medicines Questionnaire (BMQ) is a validated tool for evaluating patients’ beliefs about medical treatment [13]. The BMQ is organized into two 5-item scales. The Necessity scale assesses perceived personal necessity for prescribed treatment, and the Concerns scale identifies concerns about potential adverse consequences of specific medications. Each item is scored on a 5-point Likert scale (from 1 = strongly disagree to 5 = strongly agree). The scores obtained from items belonging to each sub-scale are summarized, ranging from 5–25 for each scale, with higher scores representing stronger necessity or concern beliefs. Muscle pain was measured at baseline and at the end of each 7-weeks treatment period using the Short-form McGill Pain Questionnaire (SF-MPQ) [22]. The SF-MPQ comprises assessment of present pain intensity linked to various pain descriptors that includes a sensory dimension (11 descriptors) and an affective dimension (4 descriptors), each descriptor is rated on a four point Likert scale (from 0 = none to 3 = severe). We measured muscle symptom location during atorvastatin treatment, including bilateral symptoms, with Margolis pain drawings [23].

### Statistics

This study is a pre-planned sub-study of the NORCOR observational study (n = 1100) and the MUSE randomized trial (n = 71), and the data used in this study is a convenience sample from the two sources. The NORCOR study was designed to have 90% power to detect the effect of psychosocial risk factor such as HADS (assuming a 20% rate of positives) for a binary outcome with a between-group difference of 10% and an overall level is 15% [17]. In the MUSE trial, we performed sample size calculations for the key secondary outcomes: assuming a mean score of HADS-anxiety of 4.7 (SD 3.7) in the refuted SAMS group [24], the study will have 80% power (with alpha = 0.05) to detect a clinically relevant mean score of ± 2.6 in the SAMS group with a total of 70 patients (50 refuted SAMS and 20 confirmed SAMS). Descriptive variables in both samples are presented as means ± standard deviation (SD), and frequencies with numbers and percentages. Differences between the groups categorized as with or without SAMS in NORCOR sample and confirmed SAMS and refuted SAMS in MUSE sample were assessed with chi-square tests for proportions and Student t-tests for continuous variables. To address the appropriateness of using parametric methods, we assessed the distribution of the continuous variables with histograms and descriptive statistics (means, medians, standard deviations, skewness, sample size). No large deviations from normality were observed and parametric methods were considered appropriate in all cases. The level of significance was set to *p* < 0.05. All data were analysed using SPSS version 26.

## Results

Those who reported SAMS had significantly lower age and higher LDL-cholesterol than those without SAMS in the NORCOR study (Table 1). There were no differences in the prevalence of SAMS in time groups since the index event. The response rate for the questionnaire was 98% (n = 1108). There were no statistically significant differences for any of the other demographic, clinical or psychological factors between the study groups.

**Table 1:** Sociodemographic, clinical and psychological factors in patients with and without SAMS^a^ in the NORCOR study

	Without SAMS (N = 1030, 93.6%)	With SAMS (N = 70, 6.4%)	*p*-value
*Socio-demographic factors*			
Age, mean (SD)	63.2 (9.6)	60.6 (9.1)	0.03
Female gender, n (%)	218 (21)	19 (27)	0.20
Low education^b^, n (%)	735 (71)	45 (64)	0.26
Living alone, n (%)	190 (20)	11 (17)	0.57
*Clinical factors*			
Myocardial infarction, n (%)	839 (79)	57 (81)	0.68
> 1 previous coronary event, n (%)	314 (30)	23 (33)	0.58
Low Density Lipoprotein Cholesterol (mmol/L), mean (SD)	2.1 (0.8)	2.6 (1.0)	< 0.01
*Psychological factors*			
HADS^c^ anxiety score, mean (SD)	4.8 (3.7)	5.1 (3.9)	0.44
HADS depression score, mean (SD)	3.9 (3.3)	3.7 (2.9)	0.63
Type D personality, SI-sum^d^, mean (SD)	7.5 (5.7)	8.2 (4.9)	0.25
Type D personality, NA-sum^e^, mean (SD)	7.0 (5.9)	7.7 (4.9)	0.31
PWSQ^f^ worry scores, mean (SD)	38.0 (12.5)	39.2 (14.1)	0.45
Bergen Insomnia scale, mean (SD)	13.8 (10.9)	15.4 (10.7)	0.25

SD, standard deviations, ^a^SAMS, statin-associated muscle symptoms, ^b^Low education was defined as completion of primary or secondary school only, ^c^HADS, Hospital anxiety and depression scale, ^d^SI-sum, Social inhibition sumscore, ^e^NA-sum, Negative affectivity sumscore, ^f^PSWQ, Penn State Worry Questionnaire

More patients in the MUSE trial with confirmed SAMS than refuted SAMS did not use statin treatment at study start and mean LDL-cholesterol level was borderline higher in this group (Table 2). No differences in smoking, physical activity, body mass index or use of analgesics between the groups were found. The response rate for the questionnaire was 100% (n = 71). There were no significant differences in psychological factors between patients with confirmed SAMS and refuted SAMS. Patients with confirmed SAMS had a slightly weaker belief in the necessity of their statin use compared to patients with refuted SAMS, independent of earlier or present statin discontinuation. No differences in the concerns about adverse effects of statins between the groups were found.

**Table 2:** Sociodemographic, clinical and psychosocial factors in patients with confirmed and refuted SAMS^a^ in MUSE trial

	Confirmed SAMS (N = 20, 28.1%)	Refuted SAMS (N = 51, 71.8%)	*p*-value
*Socio-demographic factors*			
Age, mean (SD)	64.1 (11.0)	63.2 (8.9)	0.74
Female gender, n (%)	7 (35)	16 (31)	0.77
Low education^b^, n (%)	12 (60)	33 (65)	0.71
Living alone, n (%)	4 (20)	8 (16)	0.66
*Clinical factors*			
Myocardial infarction, n (%)	17 (85)	43 (84)	0.94
> 1 previous coronary event, n (%)	5 (25)	21 (41)	0.20
Low Density Lipoprotein Cholesterol (mmol/L), mean (SD)	2.8 (1.2)	2.3 (0.9)	0.06
Rheumatic, inflammatory disease or arthrosis, n (%)	8 (40)	18 (35)	0.71
No ongoing statin therapy at study start, n (%)	5 (25)	3 (6)	0.02
Ezetemibe, n (%)	3 (15)	13 (26)	0.34
Previous discontinuation due to side-effects, n (%)	8 (40)	28 (55)	0.26
*Psychological factors*			
HADS^c^ anxiety score, mean (SD)	4.2 (4.0)	5.4 (3.2)	0.18
HADS depression score, mean (SD)	3.3 (2.1)	4.0 (2.8)	0.31
Type D personality, SI-sum^d^, mean (SD)	5.6 (6.0)	7.3 (5.9)	0.29
Type D personality, NA-sum^e^, mean (SD)	5.0 (5.1)	6.9 (4.6)	0.14
PWSQ^f^ worry scores, mean (SD)	35.8 (11.5)	39.3 (9.0)	0.18
Bergen Insomnia scale, mean (SD)	17.4 (12.1)	17.9 (11.9)	0.88
*Beliefs about Medicines*			
Necessity of statins, mean (SD)	14.9 (2.5)	17.5 (2.3)	< 0.01
Concerns of statins, mean (SD)	14.6 (4.4)	15.8 (4.7)	0.34

SD, standard deviations, ^a^SAMS, statin-associated muscle symptoms, ^b^Low education was defined as completion of primary or secondary school only, ^c^HADS, Hospital anxiety and depression scale, ^d^SI-sum, Social inhibition sumscore, ^e^NA-sum, Negative affectivity sumscore, ^f^PSWQ, Penn State Worry Questionnaire

There were no differences in muscle symptom intensity or characteristics at baseline between patients with confirmed SAMS and refuted SAMS in the MUSE trial (Table 3). During atorvastatin treatment, bilateral muscle symptoms in the lower extremities were more frequently reported in patients with confirmed SAMS than refuted SAMS (*p* = 0.01).

**Table 3:** Muscle symptom presentation in patients with confirmed SAMS^a^ and refuted SAMS in MUSE trial

	Confirmed SAMS (n = 20)	Refuted SAMS (n = 51)	*p*-value
Baseline muscle symptom intensity (VAS-score^b^), mean (SD)	4.5 (2.7)	4.7 (2.4)	0.84
*Baseline muscle symptom characteristics*^c^			
Sensory dimension, mean (SD)	10.5 (5.7)	11.5 (7.1)	0.62
Affective dimension, mean (SD)	2.9 (2.1)	2.5 (2.6)	0.58
Total sum-score, mean (SD)	13.5 (7.1)	13.8 (9.2)	0.90
*Muscle symptom location on statin treatment*^d^			
Bilateral muscle pain lower extremities, n (%)	15 (75)	21 (41)	0.01
Bilateral muscle pain upper extremities, n (%)	11 (55)	18 (35)	0.13
Bilateral muscle pain both regions, n (%)	8 (40)	9 (18)	0.05

SD, standard deviations, ^a^SAMS, statin associated muscle symptoms, ^b^VAS-score, Visual Analog Scale, ^c^Muscle symptom characteristics measured with Short-form McGill Pain Questionnaire, ^d^Muscle symptom location measured with Margolis pain drawings

## Discussion

The present study revealed that during statin treatment, symmetrical lower extremities muscle symptoms were more prevalent in patients with confirmed SAMS than in those with refuted SAMS. We did not observe any significant differences in psychological factors related to the presence of self-perceived SAMS or to the presence of refuted SAMS. This is the first study to evaluate differences in psychological factors between coronary patients with and without confirmed SAMS, under placebo-controlled conditions. Indeed, levels of these psychological factors were also similar in those with and without self-perceived SAMS. Even though a type II error due to limited number of patients cannot be ruled out, our data indicate that psychological distress does not seem to be associated with self-perceived SAMS or refuted SAMS in CHD patients. Patients with psychological distress in terms of anxiety may have increased pain perception [4]. No associations, however, were found between SAMS and anxiety symptom intensity, although pain has been associated with anxiety in primary care patients [25]. Furthermore, we did not find any relationship between level of depression symptoms and report of SAMS. Our results are in line with a randomized trial reporting that higher depression scores within the normal range at baseline did not predict changes in muscle pain severity between atorvastatin and placebo group at 6 months follow-up in patients without cardiovascular disease and depression [3]. Others have found contrasting results with higher pain scores being associated with higher scores of depression in different patient populations [25, 26].

Several reasons may explain why psychological factors were not associated with SAMS in the present study; First, it may be that our assessment instruments of psychological factors were not sensitive enough or did not assess psychological features relevant for an individual’s susceptibility to muscle symptoms in general or statin muscle symptoms in particular. Korhonen et al. found that only somatic symptoms of anxiety, but not anxiety symptoms in general, predicted non-adherence to statins [5]. However, the relationship to SAMS was not addressed. Qualitative in-depth interviews of patients with and without confirmed SAMS may possibly give additional knowledge about relevant features. Second, somatic symptoms, particularly muscle tension, are commonly found in persons with major depressive disorders and anxiety disorders, possibly explained by dysfunctions in serotonergic and noradrenergic systems [27]. These patients present higher distress scores of anxiety and depression than found in our study and the study by Zaleski et al. where patients reported scores within normal range [3]. This may suggest that in order to identify such associations, distress and associated dysfunction in biological systems may be above a certain threshold. Third, in our study, we did not have a sufficient number of patients with significant distress in order to compare those with and without psychological distress according to presence of SAMS or not.

In the present study, patients with confirmed SAMS presented highly significant more bilateral muscle symptoms in lower extremities on atorvastatin treatment compared to refuted SAMS. This accord to the STOMP study were patients on atorvastatin treatment reported predominately leg symptoms in thighs and calves, without evaluating if symptoms were symmetrical or not [28]. However, in our study, bilateral symptoms in the lower extremities also frequently occurred in patients with muscle complaints not caused by the statin. Therefore, a careful clinical interview combined with statin challenge tests, remain the recommended approach to discriminate patients with confirmed SAMS [1].

SAMS are commonly known from news in media and research presentations mainly based on registries and observational studies, with a range of estimated prevalence amounting from 7–29% [1, 29]. We found a prevalence of reported SAMS in the NORCOR sample of 6.4%. Our finding is thus in the lower rage of what has been reported in international studies and about similar to the result reported in the MUSE trial (9.9%) conducted in a coronary population from the same catchment area as that of the NORCOR study [12]. Among patients with self-perceived SAMS in the MUSE trial, the prevalence of confirmed SAMS was only 28% [12]. In the remaining 72%, the muscle complaints were of other origin, including expectation bias after initiation of statin treatment, called the nocebo effect. It is known that negative expectations and scepticism for statins is a strong predictor of later perception of side-effects [30]. This phenomenon was recently confirmed in a British study were up to 90% of statin side-effects were explained by the nocebo effect [10].

We found that patients with confirmed SAMS had weaker beliefs in the necessity of statins than those with refuted SAMS, whereas no differences between the groups in concerns about side-effects of statins were found. In an observational study in patients after myocardial infarction, the mean necessity and concern values from BMQ were comparable to the values for our total population [31]. A Swedish study reported that high perceptions of necessity to statins were associated with adherence, as expected, but the relationship to SAMS was not assessed [32]. To our knowledge the relationship between SAMS and beliefs in the necessity of statin treatment has not previously been examined. As we cannot exclude that our result has occurred by random, further research is needed to clarify the relationship between SAMS and beliefs about medication.

### Limitations and strengths

In the NORCOR observational study SAMS were categorized based on a qualitative assessment of the symptoms reported. Therefore, the true prevalence of SAMS may be slightly higher than reported. It is, however, unlikely that this will interfere with the association to the psychological variables. In the MUSE trial, we cannot preclude the possibility of classifying refuted SAMS as confirmed SAMS, due to the nocebo-effect or fluctuations in statin-independent muscle symptoms [12]. The selection of CHD-patients with reported SAMS in the MUSE study may possibly be representative for other CHD patients using statins, since the population was comparable to the NORCOR population regarding clinical, demographic and psychological characteristics and since the level of psychological factors was within the normal level. It may however be that the prevalence of these characteristics differs in other countries and populations, as for instance in statin naïve patients.

## Conclusions

Patients with confirmed SAMS more often present with bilateral lower extremities muscle symptoms during statin treatment compared to patients with refuted SAMS. We did not find any associations between psychological distress, personality, insomnia and SAMS. A careful pain history and a search for alternative causes of muscle symptoms are likely to promote communication in patients with SAMS, and may thus reduce the risk for statin discontinuation.

## Abbreviations

BMQ: The Beliefs about Medicines Questionnaire
CHD: Coronary heart disease
DS-14: Type D distressed personality Scale
HADS: The Hospital Anxiety and Depression Scale
MUSE-trial: The MUscle Side-Effects of atorvastatin in coronary patients trial
NORCOR-study: The NORwegian CORonary prevention study
PSWQ: The Penn State Worry Questionnaire
SAMS: Statin-associated muscle symptoms
SF-MPQ: The Short-form McGill Pain Questionnaire
VAS: Visual-analogue scale

## Declarations

### Availability of data and materials

The datasets generated and analysed during the current study are not publicity available due to: According to Norwegian legislation, the Norwegian Data Protection Authority, and the Committee of Ethics, we are not allowed to share original study data publicly. However, the essential data are available from the corresponding author on reasonable request.
